# Measurement of Water Velocity in Gas–Water Two-Phase Flow with the Combination of Electromagnetic Flowmeter and Conductance Sensor

**DOI:** 10.3390/s20113122

**Published:** 2020-05-31

**Authors:** Qiu-Yi Yang, Ning-De Jin, Lu-Sheng Zhai, Ying-Yu Ren, Chuang Yu, Ji-Dong Wei

**Affiliations:** School of Electrical and Information Engineering, Tianjin University, Tianjin 300072, China; qyyang@tju.edu.cn (Q.-Y.Y.); lszhai@tju.edu.cn (L.-S.Z.); renyingyuee@tju.edu.cn (Y.-Y.R.); yuchuang@tju.edu.cn (C.Y.); jdwei@tju.edu.cn (J.-D.W.)

**Keywords:** gas-water two-phase flow, electromagnetic flowmeter (EMF), instrument factor, conductance sensor, water superficial

## Abstract

A method to measure the superficial velocity of the water phase in gas–water flow using an electromagnetic flowmeter (EMF) and rotating electric field conductance sensors (REFCSs) is introduced in this paper. An electromagnetic flowmeter instrument factor model is built and the correlation between electromagnetic flowmeter output and gas holdup in different flow patterns are explored through vertical upward gas–water flow dynamic experiments in a pipe with an inner diameter (ID) of 20 mm. Water superficial velocity is predicted based on pattern identification among bubble, churn, and slug flows. The experimental results show that water superficial velocity can be predicted fairly accurately for bubble, churn, and slug flows with a water cut higher than 60% (absolute average percentage deviation and absolute average deviation are 4.1057% and 0.0281 m/s, respectively). The output of the electromagnetic flowmeter is unstable and invalid in slug flows with a water cut below 60% due to the non-conducting gas slug is almost filling the pipe. Therefore, the electromagnetic flowmeter is not preferred to be used in such conditions.

## 1. Introduction

Gas–water two-phase flow widely exists in petroleum industrial production processes. Accurately predicting flow parameters, such as superficial velocity, is of great significance for dynamic monitoring in oil fields and oilfield development. Due to the non-uniformity of gas–water two-phase flow velocity profiles and dispersed phase distribution, it is still a great challenge to measure flow parameters accurately. The electromagnetic flowmeter (EMF) is widely used to measure flow rate due to its unique advantages: simple structure, high accuracy, and strong resistance to abominable measurement conditions [1,2,3,4,5]. It shows good performance in single phase and uniform gas–water two-phase flows. As for asymmetric flow patterns, flow pattern modulation is always adopted to transform flow patterns into uniform bubble or symmetrical annular flows so that EMF can achieve a high measurement accuracy. 

In the application of EMF and the concept of weight function was proposed by Shercliff [6], and he also pointed out how mean velocity is related to EMF output voltage in uniform magnetic fields. Bevir [7] extended the weight function to three dimensions and concluded the relationship among EMF sensitivity, conductivity distribution, and velocity profile distribution. After that, Wyatt [8] analyzed EMF measurement characteristics of uniform magnetic fields on the assumption that the flow structure of bubble and annular flows are symmetrical, based on the theory proposed by Bevir [9,10]. This assumption was proved to be within 5% error by Mi et al. [11] and they also considered that this correlation of EMF in symmetrical flows (bubble/annular flow) can be applied to slug flows, which can be viewed as a combination of bubble flow and annular flow. To reduce the effect of the distribution of the gas phase on EMFs, Yang et al. [12,13] used the phase-isolation method to change the inlet flow pattern into a uniform and symmetrical flow before gas–liquid flow measurements.

Bernier et al. [14] proved that the results from EMFs represented the average velocity of the continuous liquid phase with electrical conductivity and figured out the correlation between EMF output and gas holdup in uniform two-phase flows. Cha et al. [15] proposed the sensitivity coefficient of EMFs for non-uniform but isotropic suspensions. Besides, EMF output is proved to deviate from the correlation Δ*V_TP_* = Δ*V_SP_*/(1−*Y_g_*) by the measurement system of EMFs and differential pressure sensors, when gas holdup is higher than 0.3. Considering that the output of EMFs in gas–liquid flow is affected by the flow pattern and velocity distribution [16], Deng et al. [17] establish a flow rate model of EMFs to perform liquid flow rate measurements in slug flows. Xu et al. [18] pointed out that the flow rate obtained by the EMF should be corrected, considering the slip velocity and the flow pattern by introducing a homogeneity factor. Wang et al. [19] performed an EMF–EIT data fusion for measuring continuous-phase velocity with an input of the EIT mean volume fraction. 

The measurement accuracy of EMFs is affected by the conductivity distribution and axisymmetric velocity profile distribution in gas–water two-phase flows. Without considering the effects of flow pattern, previous research modified the results of EMFs by an instrument factor model. Therefore, it is a great challenge to establish EMF instrument factor models in different flow patterns. Considering flow-concentrating measurements in oil well production, the output characteristics of an EMF in 20 mm pipes is studied in this paper. Vertical upward gas–water flow dynamic experiments are performed based on the combination system of an EMF and conductance sensors. Firstly, water holdup in different flow conditions is extracted by rotating electric field conductance sensors (REFCSs). An electromagnetic flowmeter instrument factor model is built and the correlation between electromagnetic flowmeter output and gas holdup in different flow patterns is explored. Finally, water superficial velocity is accurately predicted.

## 2. Experimental Facility and Measurement System

**Figure 1:**
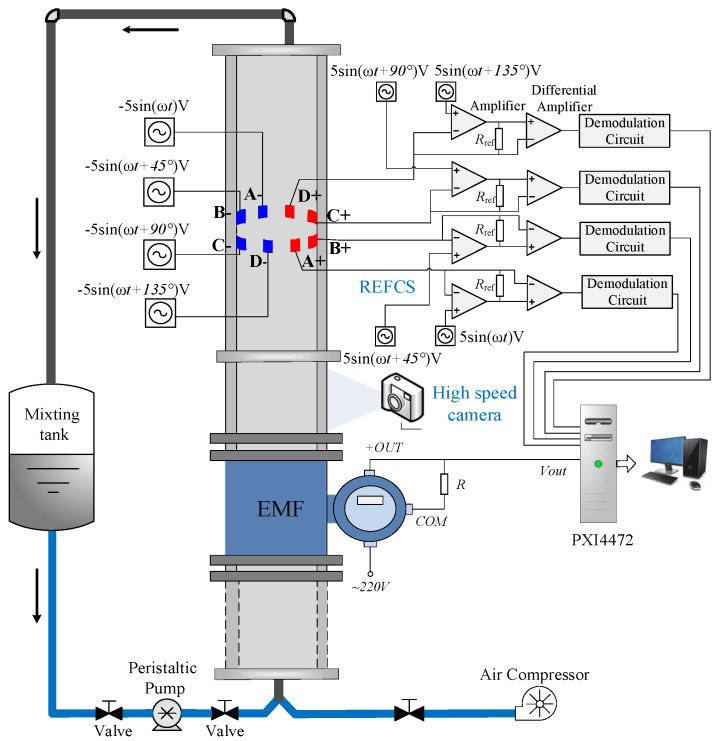
EMF–REFCS measurement system for gas–liquid two-phase flow.

The vertical upward gas-liquid two-phase flow dynamic experiments were carried out through an acrylic pipe with a 20 mm inner diameter (ID). The experimental setup is shown schematically in Figure 1. It is mainly composed of a water tank, peristaltic pumps, air compressor, metal float flowmeter, rotating electric field conductance sensor (REFCS), high-speed camera, electromagnetic flowmeter (EMF), check valve, and a vertical pipe with a length of 2.5 m. The peristaltic pumps (Model WT300F) used in the experiment are produced by Baoding Lead Fluid Technology Company. The measurement uncertainty of the industrial peristaltic pumps is equal to ±0.2%. Tap water and air are mixed through the “Y” inlet and flow into a vertical pipe. The density of water is 1000 kg/m^3^, the viscosity of water is 1 mPa·s. When the mixture fluid flows through the REFCS and EMF, measurement signals are acquired. Then the mixture fluid flows into the water tank (300 L) for recycling and air is discharged into the atmosphere. Peristaltic pumps and float flowmeters are used to control the inlet velocities of the water and gas phases, respectively. An REFCS is used to provide water holdup and recognize flow patterns. A high-speed camera is used to capture flow images in different flow patterns. An EMF is used to measure water superficial velocity in the gas–water flow.

The gas–water dynamic experiments cover three typical flow patterns: bubble flow, churn flow, and slug flow. In the experiment, the water superficial velocity *U_sw_* ranges from 0.037 m/s to 1.178 m/s and the gas superficial velocity *U_sg_* ranges from 0.055 m/s to 0.590 m/s. The signals of the REFCS and EMF are collected by the PXI-4472 synchronization card of the NI Company. The sampling frequency and sampling time are set as 2 kHz and 30 s, respectively.

### 2.1. Measurement Characteristics of the Electromagnetic Flowmeter

When a uniform fluid with electrical conductivity flows through the EMF, Shercliff [6] indicated that the potential difference Δ*V_SP_* between two point electrodes is expressed as:

(1) $$\Delta V_{SP}=\frac{Bd}{A}Q_{w}=BdU_{w}.$$

where *B* is the magnetic flux density, *d* is the distance between the two electrodes (that is, the ID of the pipe), *A* is the cross-sectional area of the pipe, *Q_w_* is the flow rate of the conductive liquid, and *U_w_* is the average velocity of the conductive liquid in the pipe cross-section.

**Figure 2:**
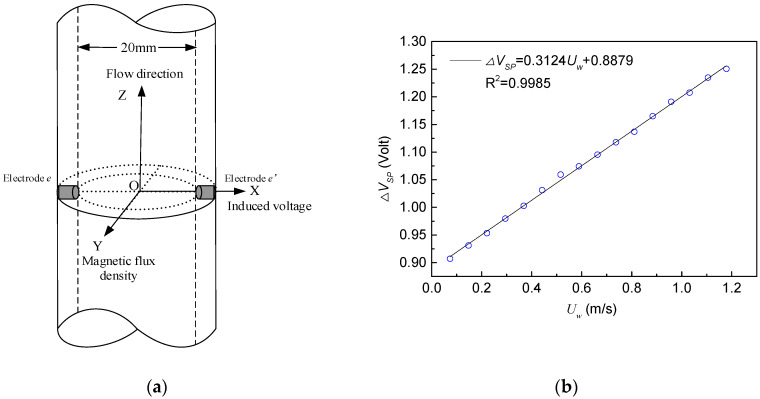
Measuring principle of the EMF. (**a**) Structure of the EMF; (**b**) calibration curve of the EMF in a water single-phase flow.

The range of the EMF (as shown in Figure 2a) used in the dynamic experiments is from 0.05 to 11.48 m/s and the accuracy is 0.3%. Figure 2b presents a good linear relationship between the voltage of the EMF and the water superficial velocity. Therefore, the correlation between the voltage of the EMF and the water superficial velocity can be expressed as follow:

(2) $$\Delta V_{SP}=k_{0}U_{w}+b.$$

By linear fitting the voltage of the EMF and the water superficial velocity, we can obtain:

(3) $$\Delta V_{SP}=0.3124U_{w}+0.8879.$$

**Figure 3:**
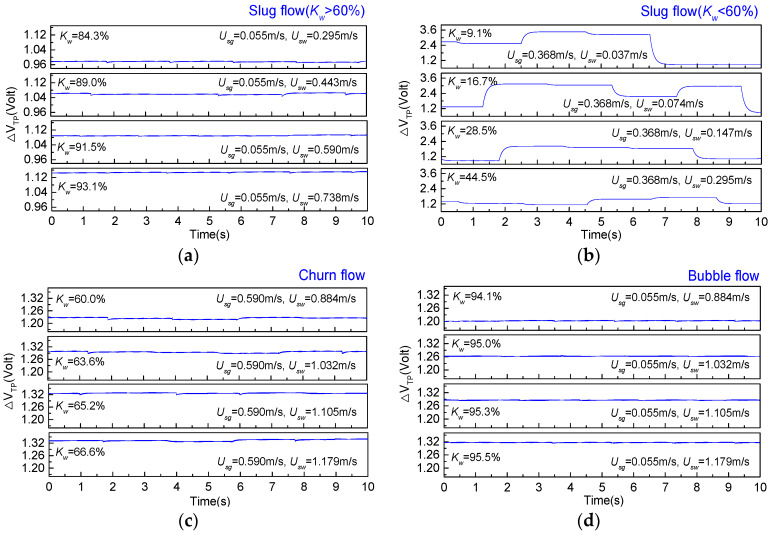
Output signals of the EMF for gas–water two-phase flows. (**a**) slug flow (*K_w_* > 60%); (**b**) slug flow (*K_w_* < 60%); (**c**) churn flow; (**d**) bubble flow.

The EMF can well reflect velocity of the conductive fluid. In this paper, the EMF is carried into the dynamic experiments of the vertical upward gas–water flow. The conductive fluid cuts magnetic lines of force when flowing through the EMF. The EMF generates an induction electromotive force. For gas–water two-phase flows, the water phase is the only conductive fluid, and the gas phase is the insulating fluid. Therefore, when the velocity of the conductive medium in the mixture fluid is different, the ability to cut magnetic field lines is different, and the EMF will produce different voltages. Figure 3 shows the output signals of the EMF for gas–water flow in different flow patterns.

The output voltages of the EMF in a gas–water slug flow are presented in Figure 3. A slug unit consists of a gas slug and a liquid slug [11]. The gas and liquid slugs alternately pass through the EMF. For slug flows with a water cut (*K_w_*) higher than 60%, the output signals of the EMF are shown in Figure 3a. The output voltage of the EMF is stable, and the voltage amplitude increases with the increase in the water superficial velocity. The output signals of the EMF in the slug flow with a water cut lower than 60% are presented in Figure 3b. For slug flows with a large gas–water ratio, the insulating gas slug almost occupies the whole space of the pipe and is connected to the pipe wall through a thin water film. As shown in Figure 3b, the amplitudes of the EMF output voltage produce large fluctuations. As the gas–water ratio increases, the amplitude of the EMF output voltage fluctuates more severely. Therefore, the study of the EMF measurement characteristics in this paper ignores slug flows with a water cut lower than 60%.

As the mixture velocity of the gas–water flow increases, the mixture flow presents a churn flow. Deformed large bubbles randomly appear in the mixture flow [20]. Unlike the intermittent flow structure of a slug flow, the churn flow has no gas slugs that almost occupy the whole space of the pipe. Though the churn flow has irregular and random phase interfaces, the water phase moves upward as a continuous phase, cutting the magnetic field line in the pipe. The output signals of the EMF, as shown in Figure 3c, present as relatively gentle and steady. As the water superficial velocity increases, the voltage of the EMF increases. As the gas-water ratio decreases, the gas-water flow pattern transforms into a bubble flow. A bubble flow is the homogeneous mixture flow with a continuous water phase, and small gas bubbles flow randomly in the continuous water phase [21]. As shown in Figure 3d, the EMF output voltages in bubble flows are very stable. Besides, as the water superficial velocity increases, the output voltage of the EMF increases.

### 2.2. Conductance Sensor for Water Holdup

**Figure 4:**
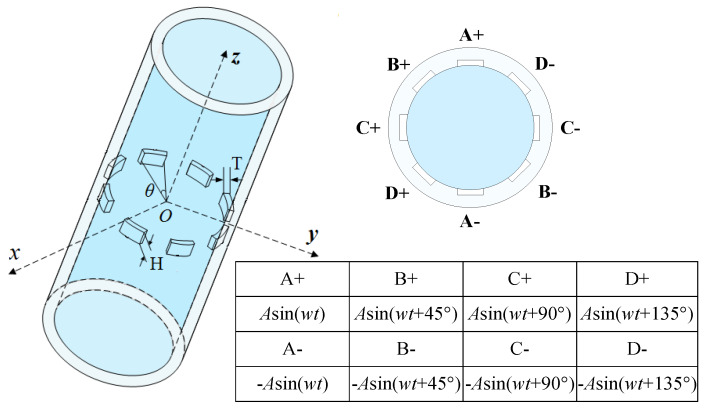
Geometric structure and parameters of the rotating electric field conductance sensor (REFCS).

A rotating electric field conductance sensor (REFCS), as shown in Figure 4, is widely used in water holdup measurements due to its quick response and high accuracy [22,23]. The REFCS is composed of four pairs of electrodes homogeneously distributed on the inner pipe wall in the same cross-section, and the field angle *θ*, axial height *H*, and radial thickness *T* of the electrodes are 22.5°, 4 mm, and 1 mm, respectively [23].

**Figure 5:**
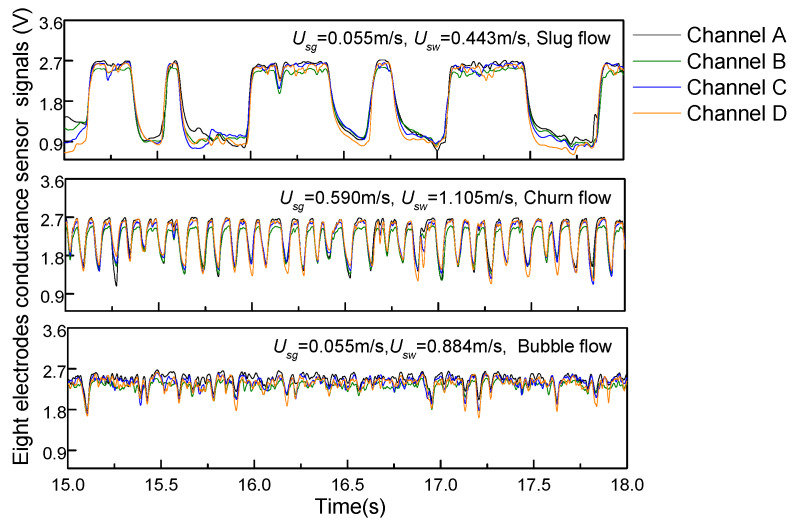
Output signals of the REFCS in gas–water two-phase flows.

Figure 5 shows the signal fluctuation of the REFCS for three typical flow patterns. For slug flows, the signals of the REFCS alternates between high and low periodically. When the gas slug flows through the REFCS’s sensitive cross-section, this corresponds to a low voltage level. On the contrary, when a liquid slug flows through the REFCS’s sensitive cross-section, this corresponds to a high voltage level. Fluctuations of a high voltage level correspond to small gas bubbles. When the mixture flow transforms to churn flow, the signals of the REFCS has periodicity similar to that of the slug flow, but the duration of the low voltage level becomes short and the amplitude is unstable. It corresponds to large deformed bubbles in the churn flow. The strong turbulent energy of the churn flow leads to coalescence, collision, and the deformation of bubbles, which corresponds to a sharp fluctuation of the REFCS signals. When the gas–liquid ratio is small, the gas–water two-phase flow presents as a bubble flow. The gas phase is broken into small bubbles by the continuous water phase and are randomly distributed in water phase, which corresponds to the signals of the REFCS randomly fluctuating within a small amplitude range.

The electrical conductivity of gas–liquid mixture flows is determined by water holdup. So, the water holdup can be obtained by an REFCS with an electrical conductivity measurement. The theory of mixture conductivity is proposed by Maxwell [24], who propounded in *A Treatise on Electricity and Magnetism* that the conductivity of a continuous phase is σ_2_, while the dispersed phase consists of small spheres with a conductivity of σ_1_. The ratio of the total volume of spheres to that of the mixture is *ϕ*, and the distance between every two spheres is far more than their radius. We assume that the gas phase is broken into small bubbles and distributed homogeneously in a continuous water phase. The mixture conductivity (*σ_m_*) with homogeneous distribution is expressed as:

(4) $$\sigma_{m}=\frac{2\sigma_{2}+\sigma_{1}-2\phi (\sigma_{2}-\sigma_{1})}{2\sigma_{2}+\sigma_{1}+\phi (\sigma_{2}-\sigma_{1})}\sigma_{2},$$

The oil phase is insulated with conductivity almost equal to zero. Equation (4) can thus be simplified as:

(5) $$\sigma_{m}=\frac{2-2\phi }{2+\phi }\sigma_{w},$$

where *σ_w_* represents water conductivity.

Substituting water holdup *Y_w_* = 1 − *ϕ* into Equation (4), the relationship among water holdup (*Y_w_*), mixture conductivity (*σ_m_*), and water conductivity (*σ_w_*) can be expressed as:

(6) $$\frac{\sigma_{m}}{\sigma_{w}}=\frac{2Y_{w}}{3-Y_{w}},$$

When the mixture fluid flows through the REFCS, the output signal is expressed as *V_m_*. *V_w_* presents the output signal of REFCS when only the water phase flows through the sensor. The normalized conductivity $G_{e}^{*}$ can be calculated:

(7) $$G_{e}^{*}=\frac{V_{m}}{V_{w}}=\frac{\sigma_{m}}{\sigma_{w}},$$

As for the REFCS, the normalized conductivity $G_{e}^{*}$ expression is as follows:

(8) $$G_{e}^{*}=\frac{1}{4}\left(G_{e}^{A}+G_{e}^{B}+G_{e}^{C}+G_{e}^{D}\right),$$

where $G_{e}^{A}$, $G_{e}^{B}$, $G_{e}^{C}$, and $G_{e}^{D}$ are the normalized conductivity of four pairs of electrodes, respectively.

Combining Equations (6) and (7), the water holdup model based on the Maxwell equation is:

(9) $$Y_{w}=\frac{3}{1+2V_{w}/V_{m}},$$

Inevitable error will be caused when the Maxwell equation, based on homogeneous fluid, is applied in inhomogeneous ones, such as slug and churn flows. Another model, proposed by Wang et al. [25], works well for slug and churn flows:

(10) $$Y_{w}=a\frac{3}{1+(2/G_{e}^{*})}+b{\left(G_{e}^{*}\right)}^{1.5016}.$$

where *a* and *b* represent the weight of the liquid and gas slugs in slug units, respectively. 

**Figure 6:**
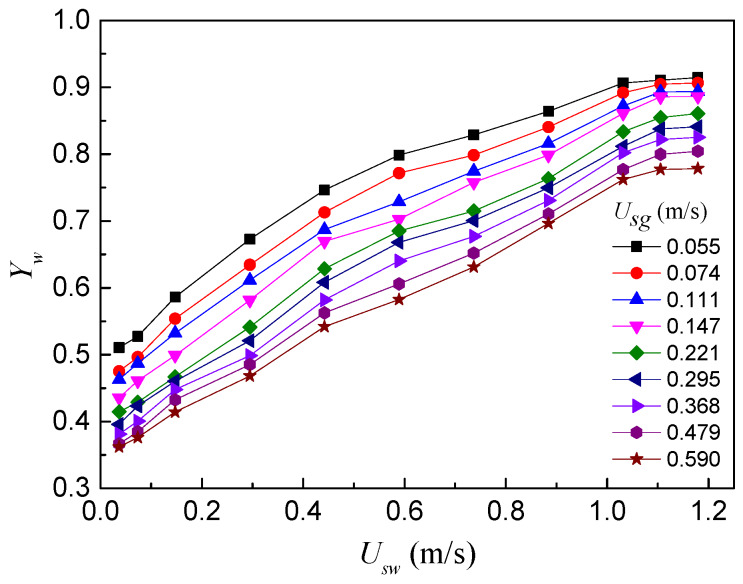
Water holdup measurement results of the REFCS.

As shown in Figure 6, the measurement chart for water holdup is drawn according to the water holdup models of the REFCS. The horizontal axis is set as the water superficial velocity, and the vertical axis is set as the water holdup. It can be seen that the water holdup measured by the REFCS increases as the water superficial velocity increases when the gas superficial velocity is fixed. When the water superficial velocity is constant, the water holdup measured by the REFCS increases with the increase in the gas superficial velocity. In summary, the REFCS has a good resolution for water holdup measurements of gas–water two-phase flows.

### 2.3. Flow Pattern Visualization

**Figure 7:**
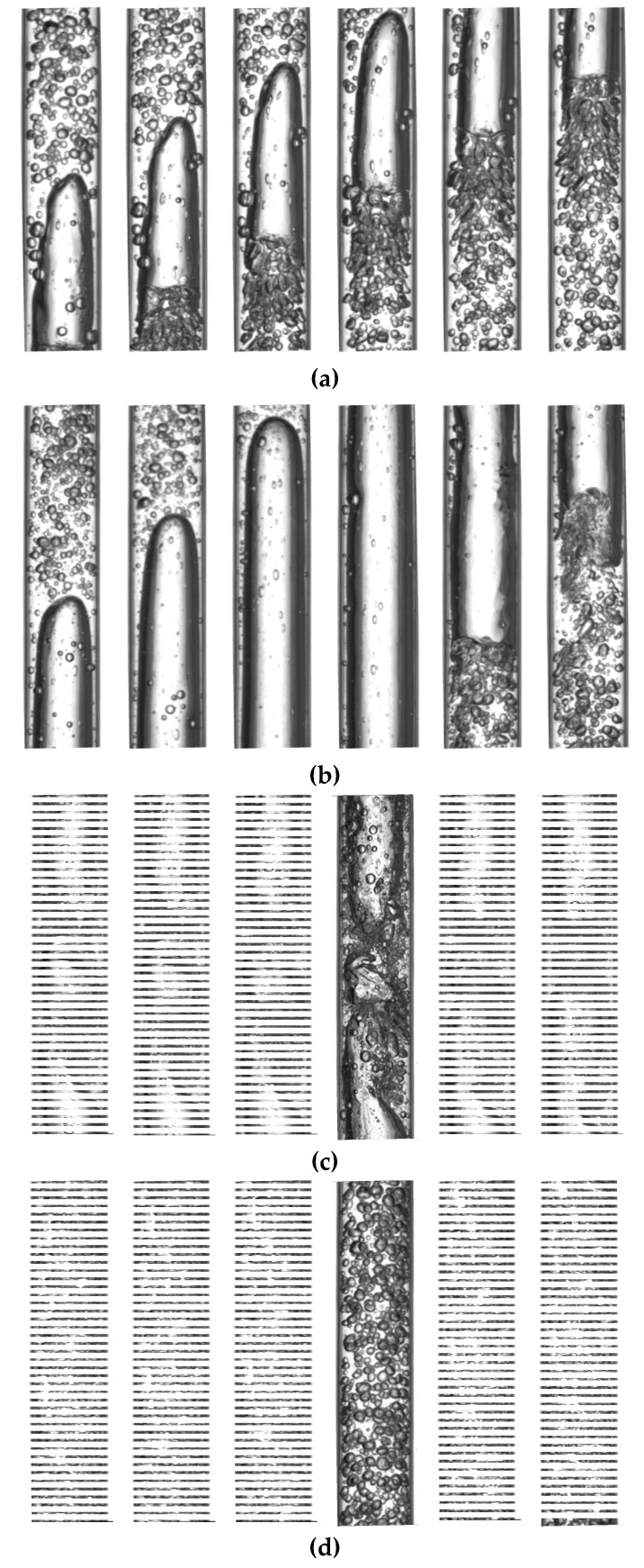
Snapshots of three typical flow patterns in gas–water two-phase flows (the interval of each frame is 0.01 s). (**a**) *U_sg_* = 0.055 m/s, *U_sw_* = 0.443 m/s, slug (*K_w_* > 60%); (**b**) *U_sg_* = 0.368 m/s, *U_sw_* = 0.295 m/s, slug(*K_w_* < 60%); (**c**) *U_sg_* = 0.590 m/s, *U_sw_* = 1.105 m/s, churn; (**d**) *U_sg_* = 0.055 m/s, *U_sw_* = 0.884 m/s, bubble.

The high-speed camera used in the gas–water dynamic experiments captured images for three flow patterns, which are slug flow, churn flow, and bubble flow. Figure 7a shows snapshots of a slug flow with a water cut higher than 60%. The liquid film connects the gas slug and pipe wall and there are random bubbles flowing in the liquid slug. The slug flow with a water cut of less than 60% is presented in Figure 7b. Gas slugs and liquid slugs alternately appear, and the gas slug almost occupies the whole space of the pipe and is connected to the pipe wall by a very thin liquid film. A large number of bubbles are observed in the liquid slug. The turbulent energy of the mixture flow is large, with a high mixture velocity, and the flow pattern evolves into churn flow, shown in Figure 7c. Large deformed bubbles and liquid slugs appear alternately, and the thick liquid film can be observed between the deformed bubbles and the pipe wall. When the gas–water ratio is small, the gas phase is broken into gas bubbles distributed uniformly in continuous water. The flow pattern presents a bubble flow, shown in Figure 7d. 

### 2.4. Flow Pattern Identification

The recurrence plot is a method to visualize the recurrence characteristics of a phase space. It is used to reveal the internal structure of a nonlinear time series, so as to give a priori knowledge of the similarity and predictability of the time series. As an effective method for two-phase flow pattern recognition [26,27], the recurrence plot was first proposed by Eckman et al. [28] in 1987.

Set an original time series as {*x*_1_, *x*_2_, *x*_3_, ·∙∙, *x*_n_}. According to Takens’s embedding theory, set the embedding dimension *m* and delay time *τ*. The time series after phase space reconstruction is:

(11) $${\vec{X}}_{i}=\left\{x_{i},x_{i+\tau },\ldots ,x_{i+(m-1)\tau }\right\},(i=1,2,\ldots ,N),$$

where *N* = *n* − (*m* − 1) *τ*. In reconstructed time series, the Euclidean norm is defined as the distance of any two elements:

(12) $$d_{ij}=\left\|{\vec{X}}_{i}-{\vec{X}}_{j}\right\|,$$

The threshold is selected as ε = α∙*std*(*x_i_*), *std*(*x_i_*) is the standard deviation of the time series after phase space reconstruction, and *α* is the empirical coefficient. Create a recurrence matrix *R_ij_* = *Heaviside*(*ε* − *d_ij_*), and the expression of the *Heaviside* function is as follows:

(13) $$Heaviside\left(x\right)=\{\begin{array}{cc} 1 & x\geq 0\\ 0 & x<0 \end{array}.$$

**Figure 8:**
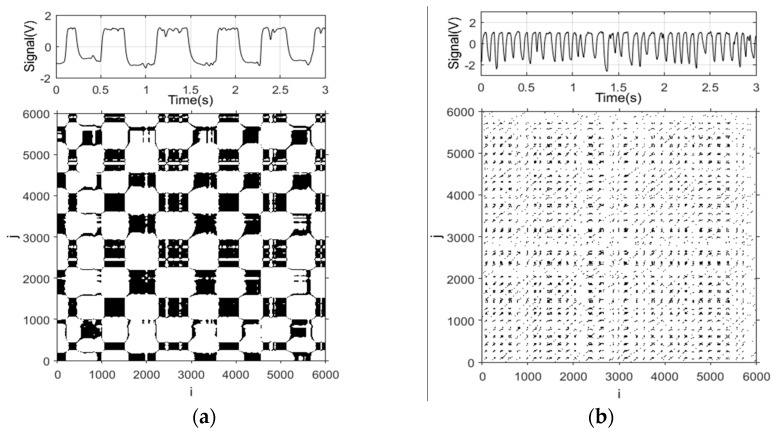

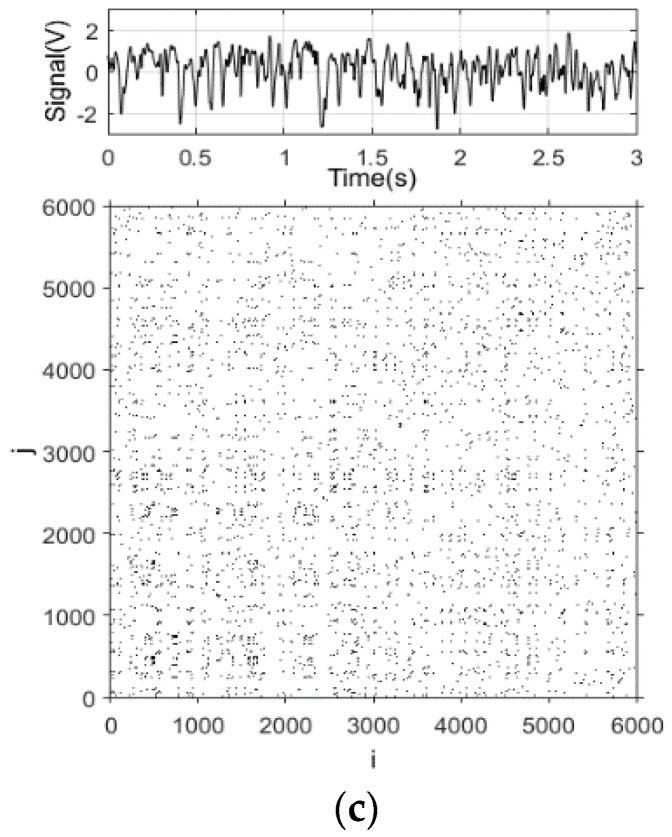
Recurrence plots under typical flow patterns of gas–water two-phase flows. (**a**) *U_sg_* = 0.055 m/s, *U_sw_* = 0.443 m/s, slug; (**b**) *U_sg_* = 0.590 m/s, *U_sw_* = 1.105 m/s, churn; (**c**) *U_sg_* = 0.055 m/s, *U_sw_* = 0.884 m/s, bubble.

Accordingly, a sphere centered at *x_i_,* with *ε* being the radius, is introduced. If *x_j_* is located in the sphere, the state is similar to *x_i_*. In this case, *R_ij_* = 1 and a point is plotted at (*i*,*j*) in a coordinate plane whose horizontal axis and vertical axis both represent the total length of the time series. By this means, the recurrence plot of the reconstructed phase space can be acquired. The signal of the REFCS is used for the recurrence plot calculation. In this paper, the value of the delay time *τ*, embedding dimension *m*, and empirical coefficient *α* are set as 2, 3, and 0.25 [20], respectively. The recurrence plots of three typical flow patterns are shown in Figure 8. 

The recurrence plot of slug flows is exhibited in Figure 8a and presents a rectangular texture structure. Regular rectangular blocks in the recurrence plot correspond to periodical flow of gas slugs and liquid slugs. The recurrence plot of churn flows, shown in Figure 8b, presents a line texture structure instead of developing into rectangular blocks. It corresponds to the large deformed bubbles not occupying the whole space of the pipe. As the flow pattern changes to a bubble flow, the texture structure shown in Figure 8c evolves into scattered points, which corresponds to the random distribution of bubbles. 

## 3. Phase Volumetric Flow Rate Determination

Bernier and Brennen [14] extended the application of EMFs to two-phase flows. They proposed a unified correlation, as presented in Equation (14) between the output voltage of an EMF Δ*V_TP_* and the gas holdup *Y_g_* when the flow pattern and slippage are neglected.

(14) $$\Delta V_{TP}=\frac{\Delta V_{SP}}{1-Y_{g}}=\frac{Bd}{A(1-Y_{g})}Q_{w}=\frac{Bd}{1-Y_{g}}U_{sw},$$

where Δ*V_SP_* represents the voltage of an EMF when single-phase water flows through. Jia et al. [29] realized the measurement of the water velocity in a gas–water flow by Equation (14). Cha et al. [16] experimentally proved that the output voltage of an EMF satisfies for Equation (14) a gas–liquid bubble flow with a gas holdup less than 0.3. 

In vertical upward gas–water flows, combine correlation Equation (3), reflecting an EMF’s output in single-phase water, with Equation (14), then we can have:

(15) $$U_{sw}=\frac{\Delta V_{TP}(1-Y_{g})-0.8879}{0.3124},$$

*Y_g_* is calculated from the water holdup measured by an REFCS (*Y_g_* = 1 − *Y_w_*). Δ*V_TP_* is the average output voltage of an EMF in a gas–water two-phase flow dynamic experiment. 

In view of the unformed distribution of a dispersed phase, the conductivity and velocity profiles in a gas–water flow and the slippage between the water phase and gas phase, Cha et al. [15] modified Equation (14) and proposed a sensitivity coefficient, based on the theory of EMFs proposed by Bevir [9]. Assuming that a non-uniform but isotropic mixture fluid flows through the EMF, the conductivity distribution (*λ*, *s*) and axisymmetric velocity profile distribution (*n*) vary with the cross-section radius *r* (0 < *r* < 1) by following a power correlation:

(16) $$\sigma (r)=1+\lambda r^{s},$$

(17) $$U\propto 1-r^{n},$$

then the output of an EMF in a two-phase flow is:

(18) $$\Delta V_{TP}=\frac{\Delta V_{SP}}{\left(1-Y_{g}\right)}\left[1-\frac{\lambda s}{\left(s+2)(s+n+2\right)}\right].$$

Considering the slippage between phases and non-uniformed distributions of conductivity and velocity, some scholars [11,12,13,18] proposed the EMF two-phase flow instrument factor *ε* (also called a homogeneity factor, a sectional shape factor of a conductive phase, etc.) related to gas holdup based on dynamic experiments. For gas–water two-phase flows, the correlation between the voltage of an EMF and gas holdup is established as follow:

(19) $$\Delta V_{TP}=\frac{1}{\varepsilon }\cdot \frac{\Delta V_{SP}}{1-Y_{g}}=\frac{1}{\varepsilon }\frac{BdU_{sw}}{1-Y_{g}},$$

According to Equation (19), the instrument factor expression of an EMF in a gas–water two-phase flow can be expressed as follows:

(20) $$\varepsilon =\frac{\Delta V_{SP}}{\Delta V_{TP}(1-Y_{g})},$$

Combining Equations (3) and (20), Equation (20) can be expressed as:

(21) $$\varepsilon =\frac{\Delta V_{SP}}{\Delta V_{TP}(1-Y_{g})}=\frac{0.3124U_{sw}+0.8879}{\Delta V_{TP}(1-Y_{g})}.$$

**Figure 9:**
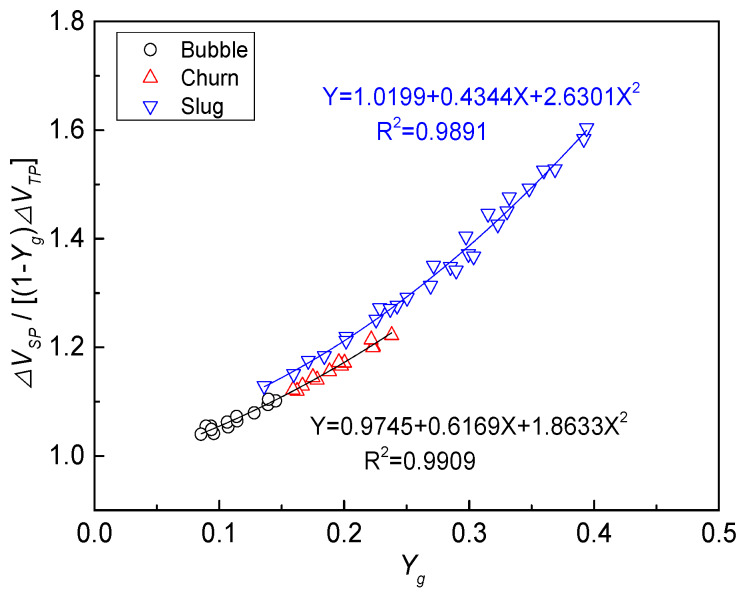
Relationship between the instrument factor of an EMF and gas holdup.

According to the instrument factor of an EMF in a gas-water two-phase flow calculated by Equation (21), the relationship between the instrument factor of the EMF and the gas holdup is drawn in Figure 9. 

It can be seen from Figure 9 that the instrument factor of an EMF in a gas–water two-phase flow increases with the increase in gas holdup. Bubbles are uniformly distributed in a continuous water phase, the slippage between phases is not obvious, and the conductivity distribution of the mixture flow is relatively uniform in bubble flow. Therefore, the EMF instrument factor of bubble flow, shown in Figure 9, is close to 1. When the flow pattern evolves into churn flow, large deformed bubbles flow in a continuous water phase. The slippage between the phases becomes obvious and the cross-section conductivity distribution is non-uniform, which corresponds to the instrument factor of the EMF gradually deviating from 1. As for the slug flow of a gas–water two-phase flow, the gas slug and liquid slug flow alternately. With the increase in gas holdup, the slippage between phases is more significant and the cross-sectional conductivity distribution of the gas slug is greater, which corresponds to the instrument factor of the slug flow moving farther away from 1. According to the fitting curve of the instrument factor with gas holdup, shown in Figure 9, correlations between the instrument factor and gas holdup under different flow patterns are as follows:

(22) $$\varepsilon =\{\begin{array}{l} 0.9745+0.6169Y_{g}+1.8633Y_{g}^{2}\;\mathrm{Bubble}/\mathrm{Churn}\;\\ 1.0199+0.4344Y_{g}+2.6301Y_{g}^{2}\;\;\;\mathrm{Slug}\; \end{array}.$$

Combining Equations (3) and (19), a correlation between the output voltage of an EMF in a gas–water flow and the water superficial velocity can be expressed as:

(23) $$U_{sw}=\frac{\varepsilon \cdot \Delta V_{TP}(1-Y_{g})-0.8879}{0.3124}.$$

**Figure 10:**
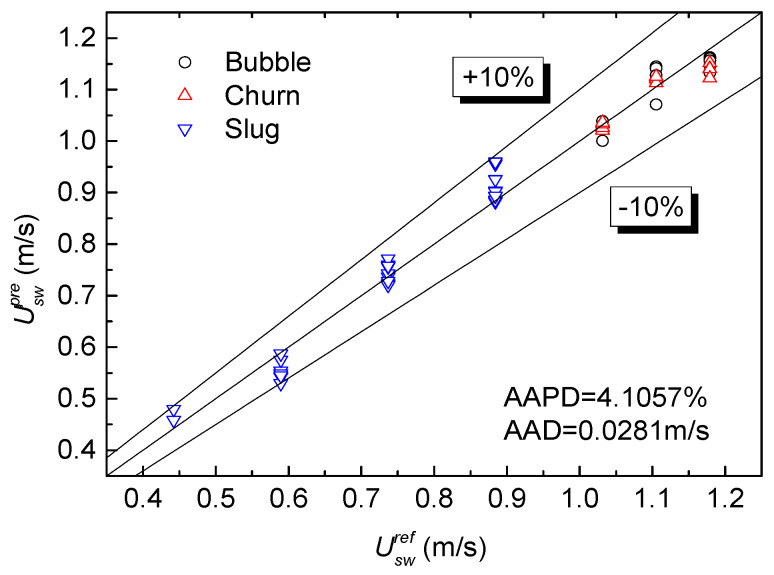
Prediction of water superficial velocity in an EMF–REFCS measurement system.

The output voltage of an EMF and water holdup measured by an REFCS are brought into Equation (23). The prediction results of the water superficial velocity under three typical flow patterns are shown in Figure 10.

To quantitatively evaluate the measurement accuracy of the water superficial velocity in a gas–water two-phase flow, two statistical indexes are introduced in this paper, i.e., the absolute average percentage deviation (AAPD) and the absolute average deviation (AAD).

(24) $$AAPD=\frac{1}{n}\sum_{i=1}^{n}\frac{\left|U_{sw}^{\exp}-U_{sw}^{ref}\right|}{U_{sw}^{ref}},$$

(25) $$AAD=\frac{1}{n}\sum_{i=1}^{n}\left|U_{sw}^{\exp}-U_{sw}^{ref}\right|.$$

where *n* presents the number of flow conditions. $U_{sw}^{exp}\;\mathrm{and}\;U_{sw}^{ref}$ present the predicted water superficial velocity by an EMF and the referenced water superficial velocity of an inlet under the i-th flow condition, respectively. 

It can be seen from Figure 10 that the AAPD and AAD of the water superficial velocity predicted by an EMF under three typical flow patterns is 4.1057% and 0.0281 m/s, respectively. It proves that the satisfactory prediction of the accuracy of the water superficial velocity can be achieved. When the water superficial velocity is predicted only in bubble and churn flows, the AAPD and AAD are 1.8635% and 0.0221 m/s, respectively. According to the images of slug flow captured by the high-speed camera, gas slug and liquid slug flows alternate and the conductive phase distributes non-uniformly. When the water superficial velocity is predicted only in a slug flow, the AAPD and AAD are 4.0007% and 0.0252 m/s, respectively. It can be seen that the EMF corrected by instrument factor can achieve a high-precision prediction for the water superficial velocity for non-uniform symmetrical flow patterns. As for the cross-correlation flowmeter studied by Wang et al. [30], the prediction of the velocity takes into account the influence of the flow pattern, but without considering the influence of water holdup on the instrument factor. The absolute average percentage error of the cross-correlation flowmeter is 7.181%. Differential pressure flowmeters are also applied in the velocity measurement of gas–water flows. Murdock [31] proposed a practical method of an orifice meter to compute two-phase flowrates without considering the effect of flow pattern. Steven [32] offered a correlation of Venturi meters for flowrate measurements in wet gas. The above two differential pressure methods are suitable for specific flow patterns and have limited adaptability under different flow patterns. The new combination measurement method in this paper establishes the EMF variable instrument factor model in different flow patterns, which increases the universality and accuracy of velocity measurements in gas–water two-phase flows.

The uncertainty of this EMF–REFCS measurement system is mainly caused by the following three reasons. Firstly, the establishment of EMF instrument factor greatly reduces, but cannot eliminate, the influence of flow pattern structure on measurement accuracy. The second reason is the gap between calibration curve of EMF in single-phase flow (seen Figure 2b) and the actual one. Thirdly, the measurement error of the water holdup measured by the REFCS is also one of the uncertain factors in the prediction of the water superficial velocity.

## 4. Conclusions

The measurement characteristics of an EMF in a gas–water two-phase flow at low velocity, dependent on flow pattern, are studied in this paper by carrying out vertical upward gas–water flow dynamic experiments. Therefore, the output model of an EMF in a gas–water flow is established to realize the prediction for the water superficial velocity with the flow pattern considered. The conclusions are as follows:

1. An EMF presents a good performance in single-phase water and uniform symmetric flow patterns (e.g., bubble flow). As it is affected by the gas phase distribution in a gas–water flow, the simple correlation of the EMF Δ*V_TP_* = Δ*V_SP_* / (1 − *Y_g_*) cannot predict the water superficial velocity in asymmetric flow patterns accurately. We find that the instrument factor of the EMF in a gas–water flow is directly affected by the flow pattern and figures out the factor under the different flow patterns in this paper. Then the water superficial velocity can be predicted accurately using the EMF, combined with the water holdup measured by an REFCS and flow pattern recognition.
2. In vertical upward gas-water two-phase flows, the EMF usually co-operates with a sensor for holdup to realize the prediction of the water superficial velocity. In this paper, a high-accuracy EMF–REFCS measurement system is built using an EMF and REFCS to predict the water superficial velocity of bubble flows, churn flows, and slug flows with a water holdup higher than 60%. The absolute average percentage deviation (AAPD) and the absolute average deviation (AAD) of the water superficial velocity are 4.1057% and 0.0281 m/s, respectively. For slug flows with a water holdup of less than 60%, the insulating phase almost occupies the whole space of the pipe, which leads to an unstable output voltage of the EMF. So, the EMF–REFCS system is not recommended for slug flows with a water holdup of less than 60%.
